# Research on Road Surface Recognition Algorithm Based on Vehicle Vibration Data

**DOI:** 10.3390/s25185642

**Published:** 2025-09-10

**Authors:** Jianfeng Cui, Hengxu Zhang, Xiao Wang, Yu Jing, Xiujian Chou

**Affiliations:** 1 State Key Laboratory of Extreme Environment Optoelectronic Dynamic Measurement Technology and Instrument, North University of China, Taiyuan 030051, China; sz202315021@st.nuc.edu.cn (H.Z.); jingyu5531@163.com (Y.J.); chouxiujian@nuc.edu.cn (X.C.); 2 Kangshuo (Shanxi) Institute for Control Residual Stress in Manufacturing, Jincheng 048000, China; wangxiao9462@163.com

**Keywords:** vehicle vibration, road recognition, 1D-CNN

## Abstract

Road surface conditions significantly impact driving safety and maintenance costs. Especially in connected and automated vehicles (CAVs), the road surface type recognition is critical for environmental perception. Traditional road surface recognition methods face limitations in feature extraction, so an improved one-dimensional convolutional neural network (1D-CNN) algorithm was proposed based on the VGG16 architecture. A vibration signal acquisition system was developed to efficiently acquire high-quality vehicle vibration signals. The optimized 1D-CNN algorithm model contains only 101.6 k parameters, significantly reducing computational cost and training time while maintaining high accuracy. Data augmentation, Adam optimization algorithm and *L*2 regularization were integrated to enhance generalization capabilities and suppress overfitting. On public datasets and actual vehicles tests, recognition accuracy rate reached 99.3% and 99.4%, respectively, substantially outperforming conventional methods. The algorithm also exhibited strong adaptability to different data sources. The research findings have implications for the accurate and efficient identification of road surfaces.

## 1. Introduction

With the continuous expansion and diversified development of highway networks, the road conditions faced by vehicles have become increasingly complex. Different pavement types have significant differences in material, structure, flatness, and other characteristics during the vehicle driving process, vehicles are subjected to irregular impacts and vibrations from the road surface while driving, which have differential effects on the degree of wear and tear on various vehicle components, causing a series of safety hazards and economic losses for vehicles [1]. Therefore, accurately identifying road surface types is crucial for ensuring vehicle maintenance and personnel safety.

At present, pavement identification methods are primarily categorized into two main categories: image-based identification methods and dynamic state-based research methods. Image-based recognition algorithms have evolved from traditional methods to those based on deep learning. Traditional methods primarily rely on manually designed features, such as texture, color, and shape, combined with machine learning classifiers (e.g., SVM and random forest) for recognition [2,3]. Although these methods perform well in specific scenarios, the feature extraction process is complex and challenging. However, its generalization ability is limited, making it difficult to adapt to complex and dynamic real-world environments. With the development of deep learning technology, methods based on convolutional neural networks (CNNs) have gradually become mainstream [4,5,6,7], which can automatically extract features and significantly improve the recognition accuracy rate and robustness. Common deep learning models include the VGG series of models, ResNet series, and MobileNet V1 [8,9,10]. Zhang et al. [11] introduced a prediction method for the forward road surface adhesion coefficient based on image recognition. They used DeeplabV3+, a semantic segmentation network, in conjunction with MobileNetV2, a lightweight convolutional neural network, to achieve road surface segmentation and road surface type identification, and they utilized lookup tables to obtain forward road surface attachment coefficients. Zhao et al. [12] proposed a road surface condition recognition method based on SVM (Support Vector Machine), segmented the original image, extracted nine dimensions of color features and four dimensions of texture features to construct a road surface feature database, and used grid search algorithms and PSO (Particle Swarm Optimization) algorithms to optimize the SVM kernel function factors and penalty factors, effectively solving the problem of road surface condition recognition in different scenarios. Pavement identification based on the dynamic state focuses on analyzing the physical response generated by the vehicle during travel on various types of road surfaces. Through an in-depth study of these physical responses, the specific type of pavement on which the vehicle is currently traveling can be accurately inferred. Yu et al. [13] proposed a CNN-LSTM model that achieves high-precision classification of seven types of road surfaces by analyzing the time series signals of three-axis acceleration and angular velocity from electric balance vehicles. This study is the first to validate the feasibility of combining low-cost Bluetooth sensors with deep learning in the context of electric balance vehicles. Wang et al. [14] proposed a comfortable driving speed strategy based on vehicle vibration signals combined with an extreme learning machine (ELM) to realize pavement recognition and use the improved ISA algorithm to generate a comfortable speed strategy, which significantly improves passenger comfort.

In the field of deep learning, despite advanced models such as transformers and self-supervised learning demonstrating strong performance in certain domains. Cao et al. [15] proposed a BiGRU and Transformer combined network architecture demonstrates superior performance in capturing short-term dependencies and dynamic changes when processing time series data compared to standalone Transformer models, thereby mitigating the vanishing gradient problem. The global hyperparameters were optimized using the Beluga Whale Optimization (BWO) algorithm, avoiding global optima and significantly improving the recognition accuracy rate and convergence speed. However, its integration of attention mechanisms, recurrent units, and optimization algorithms results in a complex structure and high computational overhead, making it unsuitable for real-time deployment. Dutta et al. [16] developed a masked autoencoder based on the vision transformer (ViT) architecture, enabling automatic road anomaly detection through self-supervised learning. Although it demonstrates significant potential in reducing reliance on labeled data, its pre-training phase incurs high computational costs, making it unsuitable for scenarios requiring rapid deployment and low latency. The overall workflow is less efficient than directly deploying lightweight supervised models, prompting numerous studies to focus on model lightweighting [17].

The above model has a large volume of input data and requires more network parameters for feature extraction and recognition, resulting in high computational complexity and long training time. In contrast, this study prioritizes deployment efficiency and practical applicability. We designed an improved 1D-CNN algorithm based on the VGG16 architecture. It was specifically designed to extract efficient features from one-dimensional vibration signals, featuring a lightweight architecture that minimizes both the number of parameters and computational overhead.

This algorithm employs optimization strategies, including data augmentation, the Adam optimization algorithm, and *L*2 regularization, to mitigate overfitting issues in vibration data. By using vibration data from various sources as input for the 1D-CNN algorithm, accurate identification of road types can be achieved, thereby validating the practicality and generalizability of the 1D-CNN algorithm.

The remainder of this paper is organized as follows: Section 2 describes the design of the vibration signal acquisition system, which effectively collects vibration signals from vehicle operation. Section 3 describes the one-dimensional convolutional neural network algorithm, based on the improved VGG16 architecture, which enhances its performance through data augmentation and algorithm optimization. Section 4 verifies the recognition performance of the 1D-CNN algorithm using public datasets and actual vehicle experiments. Finally, Section 5 draws conclusions.

## 2. Related Work

**Figure 1:**
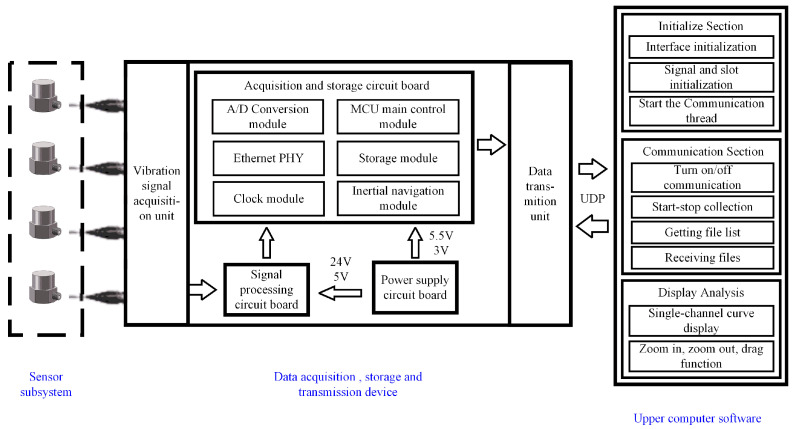
Overall architecture diagram of the vibration signal acquisition system.

To acquire vibration signals from diverse road surfaces, we designed a vibration signal acquisition system, including a sensor subsystem, a data acquisition, storage, and transmission device, and upper computer software. Using this system architecture, vibration signals from different road surfaces can be acquired efficiently and stably. The overall architecture of the vibration signal acquisition system is shown in Figure 1.

(1) Sensor subsystem

The sensor subsystem uses Integrated Electronic Piezoelectric (IEPE) sensors, which are primarily responsible for sensing road vibration signals. We chose to use the YMC121A50 model sensor from YMC PIEZOTRONICS Inc. (Yangzhou, China). Traditional piezoelectric sensors produce weak charge signals that are susceptible to interference and have limited transmission distances. IEPE sensors convert high-impedance charge signals into low-impedance voltage signals through built-in integrated circuits (such as charge amplifiers), significantly improving their anti-interference capabilities and protecting against electromagnetic interference [18]. The sensor specifications are shown in Table 1.

(2) Data acquisition, storage, and transmission device

The output signal is transmitted to the data acquisition, storage, and transmission device. The signal acquisition unit within the device converts the analog signal into a digital signal, which is then stored in the device’s data storage unit. When interacting with the upper computer software, the data acquisition, storage, and transmission device transmits the data to the upper computer software at high-speed using the UDP protocol. The main chip selection for the data acquisition device is shown in Table 2.

(3) Upper computer software

This paper developed a vibration data acquisition software based on Qt 5.14.2. The main functions used during development are shown in Table 3. The upper computer software receives and reads the vibration data, storing it in a binary format file that is highly efficient in terms of storage and fast in terms of read/write speed. It can display and analyze the received data, plot vibration amplitude curves, and monitor data characteristics.

## 3. Method

Aiming at the problems of traditional pavement recognition algorithms, such as a large volume of input data, more network parameters, high computational complexity, and long training time, this paper designs a 1D-CNN pavement recognition algorithm based on the existing classical convolutional neural network architecture VGG16 by improving the structure and parameters of the hierarchical network. The parameters of the improved neural network structure were much smaller than those of the VGG series of models. While ensuring recognition accuracy rate, it requires less computation and shorter training time. Then, the dataset was expanded using the data enhancement method to solve the overfitting problem, and the 1D-CNN algorithm was optimized by combining the Adam optimization algorithm and *L*2 regularization to handle the characteristics of vibration signals.

### 3.1. 1D-CNN Algorithm Design

#### 3.1.1. Design Principles Based on the VGG16 Architecture

**Figure 2:**
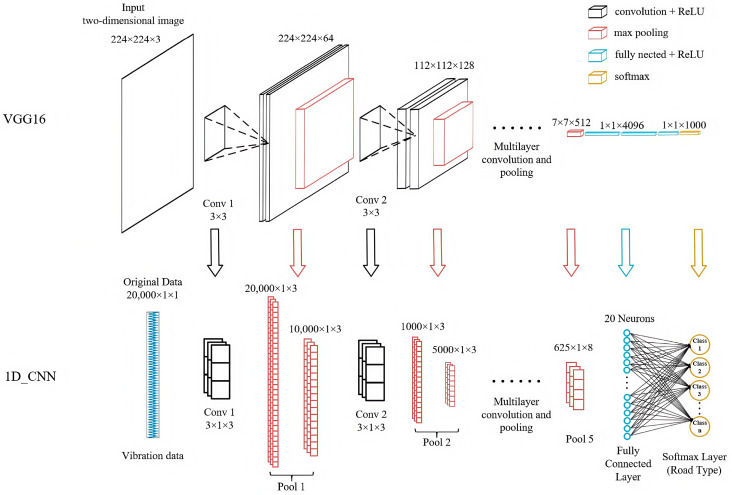
Relationship diagram of VGG16 and 1D-CNN architectures.

The structure of a convolutional neural network consists of an input layer, a convolutional layer, a pooling layer, a fully connected layer, and an output layer. The input layer receives raw data, the convolution layer extracts features using convolution kernels, the pooling layer performs a downsampling process to reduce the number of parameters and computational load, the fully connected layer integrates the extracted features, and the output layer provides classification or prediction results. The 1D-CNN algorithm designed in this paper consists of five convolutional layers, five max pooling layers, one fully connected layer, and one output layer. ReLU activation functions and Adam optimizers were used for training. The structural relationship between the VGG16 model and the proposed model is shown in Figure 2.

The figure above uses a top-down comparison layout, with the core design philosophy directly inherited from the VGG16 architecture, aiming to intuitively demonstrate the mapping relationship from the two-dimensional architecture of VGG16 to the one-dimensional architecture we proposed. Specifically manifested in the following three aspects:

(1) Core building block migration

The core innovation of VGG16 lies in stacking multiple consecutive small-sized convolution kernels (3 × 3) to replace large-sized convolution kernels, thereby reducing the number of parameters while increasing network depth and nonlinear expressive power. This design strictly adheres to this principle, designing stacked small one-dimensional convolution kernels (3 × 1) suitable for one-dimensional sequences.

(2) Macro-architecture emulation

The overall process of VGG16 can be summarized as follows: input layer, multiple levels (convolution layers + activation functions), pooling layers, fully connected layer, and output layer. Through multiple convolution layers and activation functions, higher-level features are progressively extracted. Similarly, the 1D-CNN network also employs multiple layers of one-dimensional convolutions and ReLU activation functions to progressively extract features from vibration signals. And one-dimensional max pooling layers are also introduced to reduce the sequence length, achieving the same functionality. The final output is the classification result.

(3) Continuation of design philosophy

The 1D-CNN we designed inherits VGG16’s architecture philosophy of prioritizing depth over breadth, endowing the network model with stronger representational capabilities. By employing multiple convolutional layers that progressively increase in depth (with the number of filters gradually increasing from three to eight), the design aims to gradually enhance feature complexity while reducing their length through pooling layers. This approach ensures performance while keeping the model’s parameter count significantly lower than that of VGG16.

In summary, our 1D-CNN algorithm is not merely inspired by VGG16 but represents a direct application and precise adaptation of VGG16’s core design philosophy to the field of one-dimensional signal processing.

#### 3.1.2. Algorithm Lightweight Design

As mentioned in the introduction, traditional image-based methods (such as VGG series, ResNet series, etc.) and their one-dimensional variants, though highly performant, typically face challenges such as large input data volumes, numerous network parameters, and high computational complexity, leading to prolonged training times and making deployment on resource-constrained edge devices difficult. The vehicle vibration signals addressed in this study, though one-dimensional data, have individual sample lengths of up to 20,000 data points. If a deep structure similar to VGG series is directly adopted, the computational and storage overhead would remain enormous. Therefore, one of the core objectives of this study is to thoroughly lightweight the VGG series architecture while retaining its strong representation capabilities, making it more suitable for processing one-dimensional vibration signals and meeting the requirements of potential low-power, real-time application scenarios.

Both the VGG series of models and the 1D-CNN are variants of the convolutional neural networks. The VGG series of models is commonly used for processing two-dimensional image data (height × width × channel), whereas the 1D-CNN is primarily applied to one-dimensional sequence data (length × channel). The VGG series of models constructs a deep network structure by stacking multiple small (3 × 3) convolutions, enabling it to capture more complex features. Its sliding direction is height and width, whereas 1D-CNN uses a one-dimensional convolution kernel (k × 1) with a sliding direction along the sequence length. The VGG series of models has a large number of parameters (approximately 138 million) and relies on fully connected layers, making it prone to overfitting. In contrast, the 1D-CNN structure is simple and has fewer parameters. In this study, the network has 101.6 k parameters, approximately 0.074% of the VGG16 architecture, making it suitable for lightweight tasks (such as sensor data analysis). The specific architecture, parameters, and output dimensions of the lightweight 1D-CNN are shown in Table 4.

**Figure 3:**
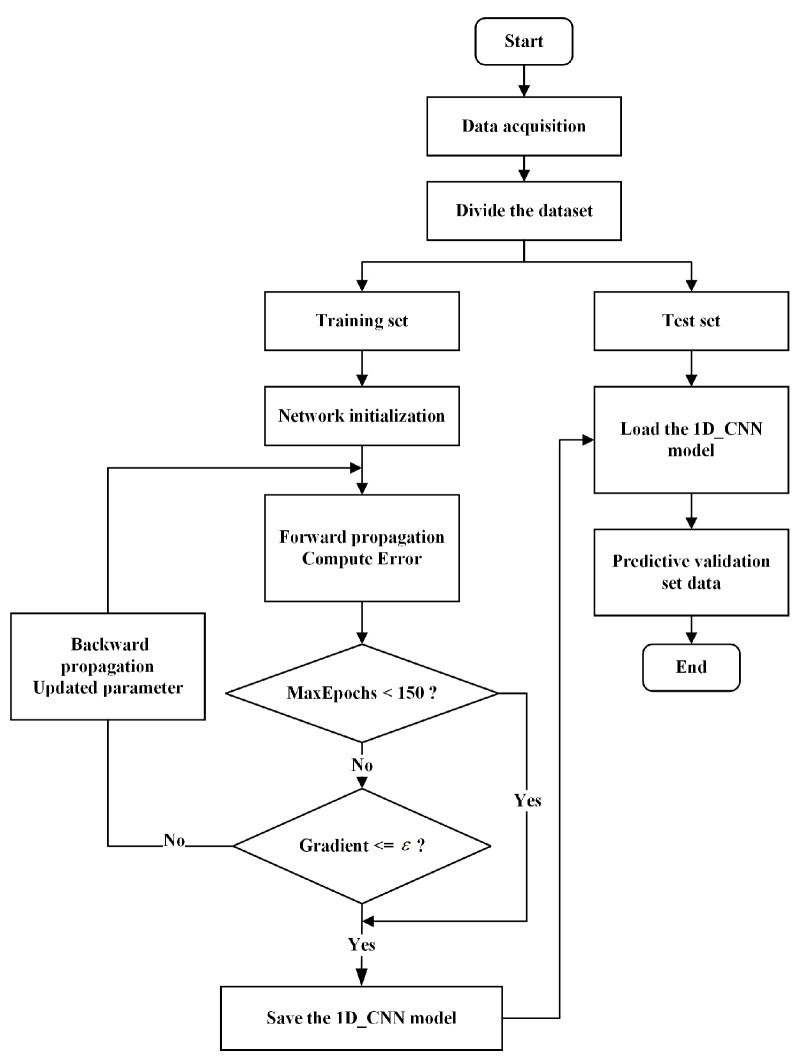
Flowchart of Road Surface Recognition Algorithm.

Therefore, a 1D-CNN based on the VGG16 architecture was used to identify several road surface types. First, vibration signals from different road surfaces were acquired, and the acquired data were preprocessed to be used as input sample data for 1D-CNN road surface recognition. After preprocessing, the signals were divided into training and test sets according to a specific ratio. In this process, the 1D-CNN uses forward propagation and backward propagation algorithms to analyze the information contained in the vibration signals and extract relevant features of different road surfaces. After feature extraction is completed, the classification layer establishes a correspondence between the time–frequency domain features and various road surfaces, enabling accurate determination of road surface types. A flowchart of the road surface recognition algorithm is shown in Figure 3.

ε is the gradient threshold, with a value of $\varepsilon =1\times 10^{-5}$.

### 3.2. Data Augmentation

**Figure 4:**
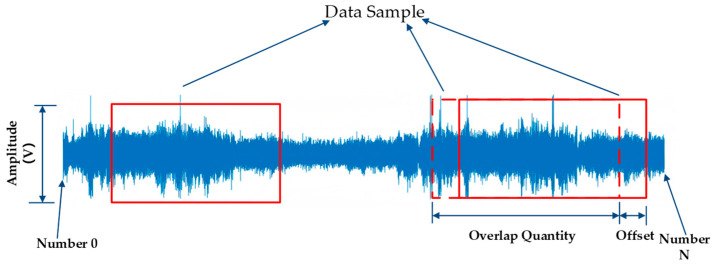
Data augmentation methods.

Data augmentation is an important technique. Its core idea is to generate new sample data through a series of transformation operations on the original dataset, thereby increasing the scale and diversity of the dataset. For one-dimensional vibration signals, this study used an overlapping sampling strategy for data augmentation. The basic principle is to sample the original data at a certain interval so that adjacent sampling windows overlap partially, thereby generating more samples from the limited original data and increasing the dataset size [19]. The overlapping sampling method is shown in Figure 4.

**Figure 5:**
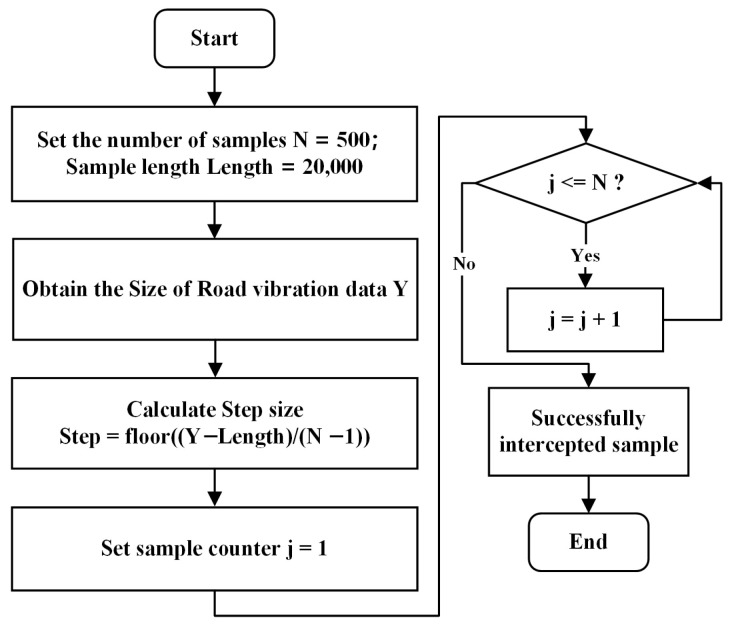
Data augmentation flowchart.

In the network, the input samples consist of 20,000 data points, the sample size for each road surface type is adjusted according to the size of the dataset. Since the data collection times vary for each type of road surface, the magnitude of the vibration signals acquired for each type of road surface also varies. To ensure consistency in the number of samples and sample length for each road surface type in the input network, the offset step size for each road surface type must be calculated based on the actual amount of data acquisition. A flowchart of the data augmentation process is shown in Figure 5, where N represents the sample size for each type of road surface, which is set to 500.

Data augmentation can increase the amount of data, providing neural networks with more training samples, which helps improve the model’s generalization ability and reduces the risk of overfitting. Since overlapping sampling preserves the local continuity of the original data while introducing some variation through different sampling positions, it enables the model to better learn the features of vibration signals across different time segments, including transient fluctuations and periodic characteristics. This enhances the model’s ability to analyze and predict vibration signals, making the trained model more practical and robust.

### 3.3. Algorithm Optimization

#### 3.3.1. Adam Optimization Algorithm

Although vibration signals are essentially one-dimensional time series, their dynamic characteristics can be mapped to high-dimensional feature spaces through time–frequency analysis, revealing significant non-stationary characteristics and noise interference. Therefore, vibration signals from different road surfaces will have sparse but important features. Using the Adam algorithm [20], the step size is dynamically updated for each parameter using an adaptive learning rate, which effectively captures both high-frequency and low-frequency features in vibration signals and avoids training instability caused by inappropriate learning rates. At the same time, its momentum mechanism reduces gradient oscillations caused by noise by accumulating historical gradient information, making training smoother. For sparse features in the vibration signals, Adam ensured that these features were fully updated, improving the classification accuracy rate.

The core idea of the Adam optimization algorithm is to combine momentum mechanisms (first-order moment estimation, accelerated convergence) and adaptive learning rates (second-order moment estimation, adaptation to different parameter gradient changes), and introduce bias correction during updates to eliminate initial bias, thereby achieving efficient and stable parameter optimization that can quickly adapt to complex or sparse gradient distributions.

(1) First and second moment estimates

The Adam optimization algorithm first calculates the first and second moment estimates of the gradient. The first-order moment estimation is the expected value of the gradient, which is an exponentially moving average of the gradient, providing a smooth gradient direction and helping to accelerate gradient descent. The second-order moment estimation is the expected value of the square of the gradient, reflecting the dispersion of the gradient, and assigning adaptive learning rates for each parameter to make them adapt to different gradient variations. The equations for the first and second moment estimates are as follows:

(1) $$m_{t}=\beta_{1}m_{t-1}+(1-\beta_{1})g_{t}$$

(2) $$v_{t}=\beta_{2}v_{t-1}+(1-\beta_{2})g_{t}^{2}$$

where $m_{t}$ and $v_{t}$, respectively, denote the first-order moment estimation and second-order moment estimation at the step t, $g_{t}$ denotes the gradient at step t, $\beta_{1}$ and $\beta_{2}$ are hyperparameters, representing the first-order moment attenuation coefficient and the second-order moment attenuation coefficient, respectively, typically taking values between zero and one.

(2) Bias corrected

When calculating the first and second moment estimates, the exponentially weighted moving average method was used, so in the initial stage, the estimate values tend to be biased toward the initial values. In order to eliminate this bias, the Adam optimization algorithm corrects the bias in the first and second moment estimates using the following equations:

(3) $${\hat{m}}_{t}=\frac{m_{t}}{1-\beta_{1}^{t}}$$

(4) $${\hat{v}}_{t}=\frac{v_{t}}{1-\beta_{2}^{t}}$$

where ${\hat{m}}_{t}$ and ${\hat{v}}_{t}$, respectively, denote the bias correction of $m_{t}$ and $v_{t}$, t denotes the current iteration step.

(3) Update parameters

After obtaining the first and second moment estimates with bias correction, the Adam optimization algorithm updates the parameters using the following equations:

(5) $$\theta_{t+1}=\theta_{t}-\frac{\alpha }{\sqrt{{\hat{v}}_{t}}+\omega }{\hat{m}}_{t}$$

where $\theta_{t+1}$ denotes update parameters, $\frac{\alpha }{\sqrt{{\hat{v}}_{t}}+\omega }$ can be regarded as the learning rate of $\theta_{t+1}$, ω is a tiny constant, usually taken as $1\times 10^{-8}$.

#### 3.3.2. *L*2 Regularization

During network training, it was observed that the model’s performance on the training set was significantly better than its performance on the test set. This phenomenon indicates that the model overfitted to the noise and details in the training data, resulting in poor generalization ability on unknown data. To solve the overfitting problem, *L*2 regularization (also known as weight decay) was introduced into the model’s loss function.

*L*2 regularization adds a penalty term to the loss function. Usually, it is the *L*2 norm of the model parameters (that is, the sum of the squares of the parameters) multiplied by a regularization coefficient. The equation is:

(6) $$J(\theta )=L(\theta )+\lambda \times {\left\|\theta \right\|}_{2}^{2}$$

where J(θ) is the loss function after regularization, L(θ) is the original loss function, θ is a parametric model, λ is the regularization coefficient, which controls the strength of weight decay, and ${\left\|\theta \right\|}_{2}^{2}=\sum_{i=1}^{n}\theta_{i}^{2}$.

## 4. Experimental Results

This paper verified that the 1D-CNN algorithm can efficiently and accurately distinguish road surface vibration data. Compared with the results of different algorithms, the 1D-CNN algorithm has better performance in road surface recognition applications. Subsequently, to verify the strong generalization ability of the designed 1D-CNN algorithm, an actual vehicle test system was set up to acquire vibration signals from various actual road surfaces. The 1D-CNN algorithm was then used for classification and recognition to evaluate the performance of the entire recognition system.

### 4.1. Algorithm Verification Using Public Datasets

#### 4.1.1. Parameter Setting

**Figure 6:**
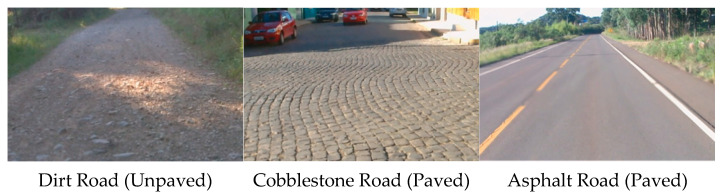
Surface types of the public dataset.

We used three publicly available datasets of road vibration signals, published by Jeferson Menegazzo on the Kaggle website, as the data source for our road identification experiment [21]. The road surface types in the public dataset are shown in Figure 6.

In this paper, to ensure the objectivity of model evaluation, a hold-out method was used to divide the road surface vibration dataset into a training set (80%) and a test set (20%). This method is implemented using the cvpartition function in MATLAB (R2023a), ensuring the independence of data distribution between the training set and the test set. Then, different road surfaces are labeled as shown in Table 5. During training, in the Adam optimization algorithm, the attenuation coefficient were $\beta_{1}=0.9$ and $\beta_{2}=0.999$; $\omega =10^{-8}$; and the maximum number of training epochs was set to 150.

#### 4.1.2. Test Results

**Figure 7:**
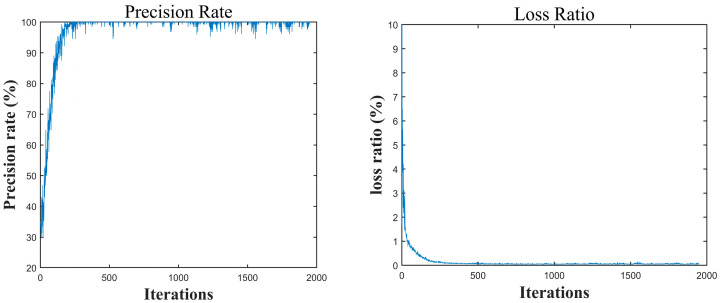
Accuracy rate and loss rate curve chart.

The test results showed that the 1D-CNN model achieved good classification performance on the test set, with an overall accuracy rate of 99.3%. During the neural network training process, the loss rate and accuracy rate were monitored, and corresponding training curves were plotted. The change curves of accuracy rate and loss rate are key visualization tools for evaluating model performance and training dynamics. The accuracy rate curve reflects the trend of classification accuracy rate changes in the training set and test set. In contrast, the loss rate curve reflects the decay of model prediction errors with the number of iterations. Ideally, the training loss rate should decrease steadily and converge synchronously in the test set, indicating that the network is effectively learning data features. A sudden increase or continuous fluctuation in loss rate often means that the learning rate is set incorrectly, or that there are problems such as gradient disappearance or explosion. Accuracy rate and loss rate are shown in Figure 7.

As shown in the figure above, the accuracy rate and loss rate exhibited distinct stage-like characteristics as the number of iterations increased. In the initial stage (0–100 iterations), the accuracy rate rapidly increases from 10% to 90%, whereas the loss rate sharply decreases from 7% to 0.5%, indicating that the model can quickly learn key features and reduce errors; In the middle stage (100–300 iterations), the changes in both metrics gradually slow down, with accuracy rate stabilizing between 98% and 100%, and loss rate remaining between 0% and 0.5%; In the later stage (after 300 iterations), both metrics stabilize with only minor fluctuations, indicating that the 1D-CNN model has achieved optimal performance.

**Figure 8:**
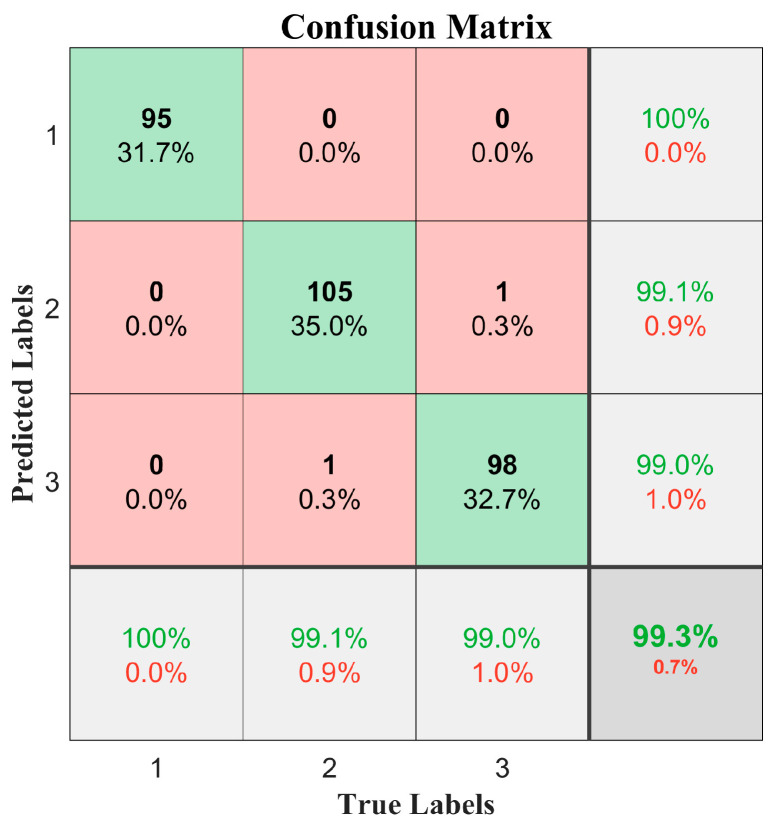
Public dataset confusion matrix results.

In classification problems, the confusion matrix is a standard analysis method used to evaluate a model’s performance in classification. By displaying the classification results for each category (including right and wrong classifications), it provides a more detailed understanding of the model’s strengths and weaknesses [22]. Specifically, the rows of the confusion matrix represent the true categories, whereas the columns represent the predicted categories. The values on the diagonal indicate the number of samples that were correctly classified, while the values off the diagonal represent incorrect classifications. By analyzing the confusion matrix, we can identify which categories are classified effectively and which categories are prone to confusion. In this experiment, the confusion matrix was used to evaluate the classification performance of the 1D-CNN model for three types of road surfaces. The confusion matrix results on the test set are shown in Figure 8. By analyzing the confusion matrix, we could thoroughly evaluate the classification performance of the model and identify confusion between different categories. The confusion matrix results showed that the three types of road surfaces were accurately identified, verifying that the 1D-CNN algorithm is well-suited for road surface recognition applications.

Jeferson Menegazzo used the collected data to identify road types, employing algorithms such as KMC, SVM, KNN, LSTM, CNN, and CNN–LSTM [23]. The performance of different algorithms is shown in Table 6.

The high accuracy rate of the 1D-CNN algorithm primarily stems from its efficient extraction of features from one-dimensional data. When processing this dataset, the sequential features are critical for classification. By designing convolutional kernels and network structures, 1D-CNN can accurately capture the local patterns and dependencies of these features, enabling precise classification decisions. Compared to traditional KMC, SVM, and KNN algorithms, 1D-CNN can automatically learn data features without the need for complex manual feature engineering, significantly improving the efficiency and accuracy rate of feature extraction.

#### 4.1.3. Performance Verification

By calculating the accuracy rate of the training and test sets separately, we can preliminarily determine that the algorithm has not overfit. To further verify the algorithm’s performance, we conducted the following three experiments to rule out overfitting.

(1) Class Imbalance Check.

The frequency table shown in Table 7 was obtained by performing a category imbalance check on the dataset. This indicates that the data distribution is uniform and that there are no category bias issues.

(2) 5-fold cross-validation experiment

To verify the generalization ability of the model, 5-fold cross-validation was used to evaluate the model. The results are shown in Table 8.

The result shows that the model achieves accuracy rate on the training set and 96.47%±2.02% accuracy rate on the test set, with a difference of 3.43%. This indicates that the model has good learning ability and generalization performance, and no overfitting issues have been observed.

(3) Noise robustness test

To assess the sensitivity of the model to input perturbations, we conducted tests with Gaussian noise of varying intensities. The results are shown in Table 9.

Noise test results show that the algorithm has good robustness. It maintains an accuracy rate of 92.67% even at a noise intensity of 20%, and only drops significantly under extreme noise conditions, proving the applicability of this method in actual noisy environments.

Based on the class imbalance check, 5-fold cross-validation, and noise robustness test results, we confirmed that the algorithm does not exhibit overfitting.

### 4.2. Actual Vehicle Testing and Verification

#### 4.2.1. Testing Plan Design

**Figure 9:**
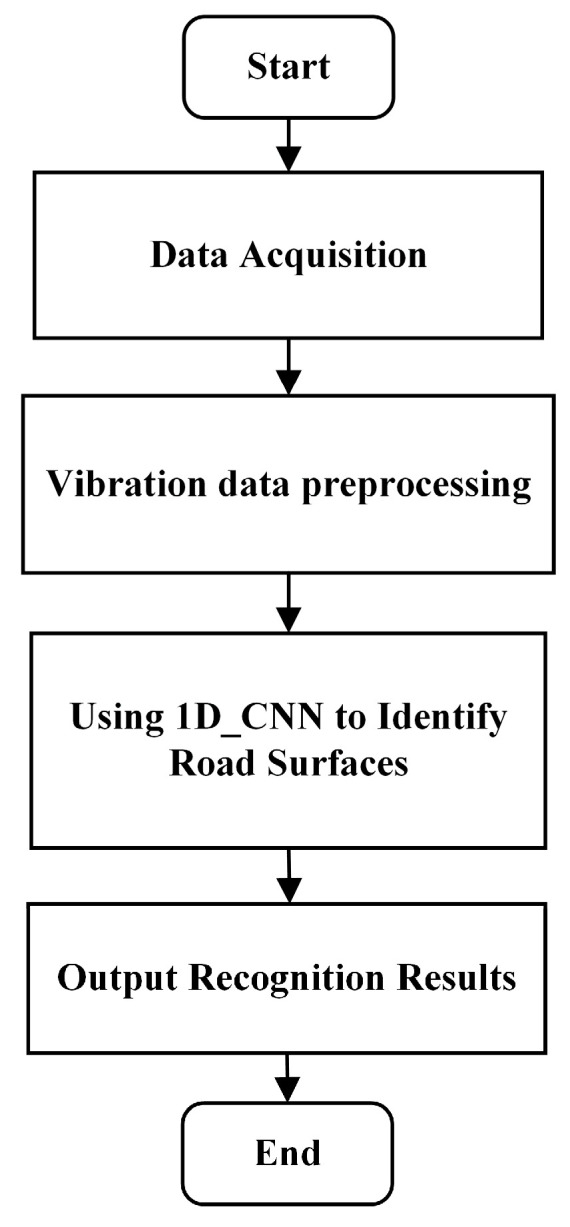
Road surface recognition implementation flowchart.

The road surface identification test plan flowchart is shown in Figure 9.

**Figure 10:**
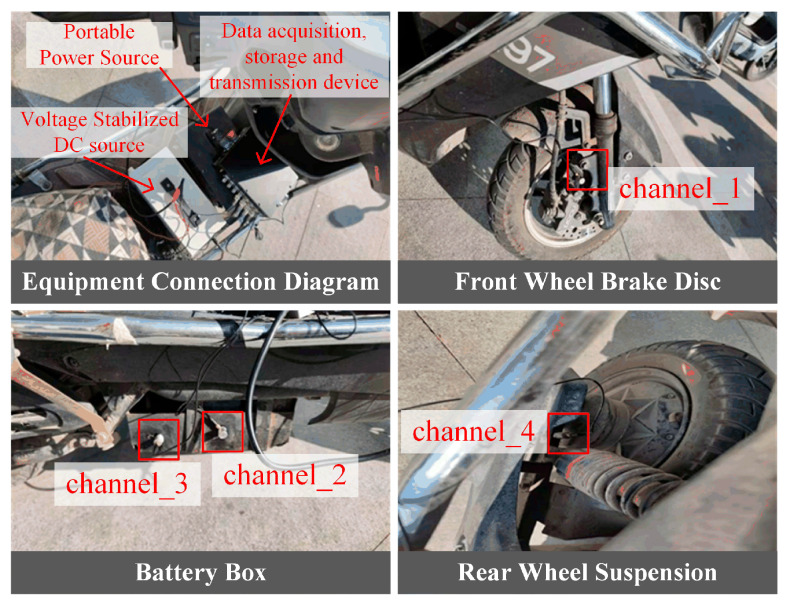
Actual vehicle test diagram.

This test used a two-wheeled electric vehicle for testing, with four channels used to collect vibration signals. Channel one was connected to the front wheel brake disc, channels two and three were connected to the battery box, and channel four was connected to the rear wheel suspension. The actual test diagram is shown in Figure 10.

When acquiring vehicle vibration signals, the battery box plays a key role in power transmission, battery protection, and overall vehicle performance, and its vibration monitoring is also crucial. Therefore, two sensors were connected to the battery box to comprehensively and accurately capture its vibration signals, ensuring the reliability and integrity of the monitoring data. We found that the two sensors exhibited essentially consistent vibration characteristics on different road surfaces; therefore, the vibration data from channel two was used for the battery box in subsequent tests.

Road surface texture characteristics and material properties jointly determine the vibration response spectrum generated when vehicles pass over the road surface. There are significant differences in the spatial frequency distribution and amplitude characteristics of various road surfaces, providing a physical basis for road classification based on vibration signals. This test selected five typical road surfaces to acquire vibration signals. The vibration data sets for five types of road surfaces, including cement road, red brick road, asphalt road, stone-paved road, and dirt road, were divided into a training set (80%) and a test set (20%). Similarly, different road surfaces are labeled as shown in Table 10.

Among these, the regular expansion joints and fine roughness on the surface of cement roads can easily cause periodic vibration responses. Red brick roads generate high-frequency vibrations due to the height differences between the bricks. Asphalt roads have smooth, dense surface characteristics and produce low-frequency, small-amplitude vibration responses. The irregular layout of stone brick roads can cause random impact vibrations for vehicles. The soft and uneven nature of dirt roads generates complex, non-steady vibration signals. By installing sensors on the brake disc, battery box, and rear wheel suspensions of electric vehicles, vibration data can be acquired simultaneously while driving on various road sections.

#### 4.2.2. Vibration Signals Acquisition

**Figure 11:**
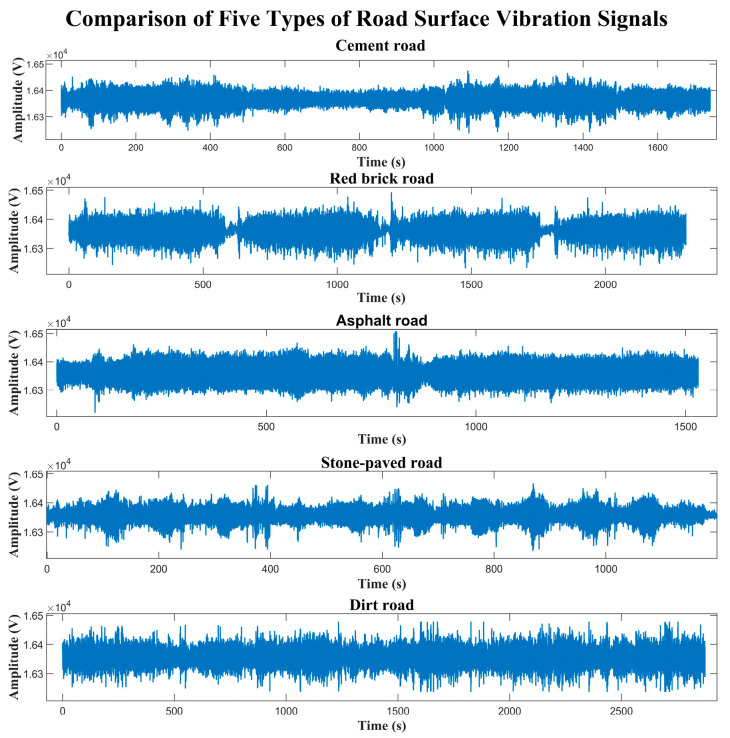
Five types of road surface vibration data.

To select the vehicle parts that play a key role in road surface recognition, we collected vibration signals from the front wheel brake disc, battery box, and rear wheel suspension on the above five types of road surfaces. For each type of road surface, record the corresponding collection time in detail. After data collection, the initially obtained road surface vibration data is split and merged to obtain complete vibration data for each type of road surface. Taking the front wheel brake disc as an example, the partial vibration raw data collected from different road surfaces is shown in Figure 11.

#### 4.2.3. Road Surface Recognition Test Verification

**Figure 12:**
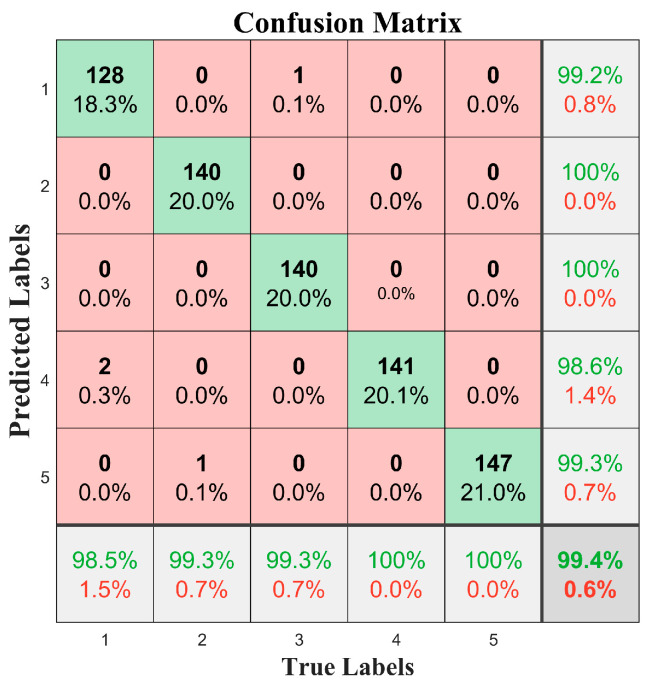
Front wheel brake disc data for five types of road surface recognition results.

For vibration signals from the above five types of road surfaces, the preprocessed vibration data were used as input for the 1D-CNN algorithm to obtain a confusion matrix of road surface recognition results. This matrix was presented in an intuitive table format to show the model’s correct and incorrect classifications of various samples. The confusion matrix for classifying and identifying five types of road surfaces using the 1D-CNN algorithm on vibration data collected from the front wheel brake discs at a speed of 30 km/h is shown in Figure 12, with an identification accuracy rate of 99.4%.

From the diagonal elements of the confusion matrix, we can see the number of samples that the model classified correctly. The recognition performances for red brick roads (label 2) and asphalt roads (label 3) were excellent. Red brick roads and asphalt roads were correctly recognized 100% of the time, indicating that the model could accurately capture their features. The accuracy rate for dirt roads (label 5) reached 99.3%, with only 0.7% misclassification as red brick roads. The analysis showed that the vibration characteristics of the two overlapped in some frequency bands. The accuracy rate for cement roads (label 1) was 99.2%, with 0.8% misclassified as asphalt roads, which was consistent with the similarity between the two types of road surfaces in terms of their low-frequency vibration responses. Stone-paved roads (label 4) had an accuracy rate of 98.6%, with 1.4% misidentified as cement roads. This was related to the fact that both were hard surfaces, which produced similar ranges of mid- to high-frequency amplitudes.

After acquiring and preprocessing vibration data from three key areas—the front wheel brake disc, battery box, and rear wheel suspension—under the above five different road conditions, the 1D-CNN algorithm was used to accurately identify different road types. The recognition accuracy rates at the same vehicle speed (10 km/h) are shown in Table 11.

During the process of receiving road surface excitation, the signal transmission path and the structural characteristics of the vehicle, including those of different vehicle components, affect the integrity and accuracy rate of the vibration signals. Road surface excitation is transmitted directly to the front brake disc through components such as tires and wheel hubs. The signal transmission path is relatively short and direct, with minimal interference from suspension filtering and body buffering, allowing for more complete retention of the high and low frequency characteristics of road surface excitation. In contrast, the battery box must pass through multiple stages, including the tires, suspension system, and body. The suspension system filters out much of the road excitation; the rear suspension must withstand both the vertical excitation from the road surface and the torque transmitted from the vehicle’s drive system, resulting in coupled interference and increased signal complexity. Therefore, the vibration data from the front brake disc most accurately reflect road surface characteristics and have the highest recognition rate.

To further validate the effectiveness of 1D-CNN in road surface recognition, we simulated the driving speed conditions of motor vehicles under different gear ratios. We acquired vibration data from the front brake disc of vehicles traveling at different speeds on five different types of road surfaces. After preprocessing the vibration data, we used the 1D-CNN algorithm for recognition, with the accuracy rates shown in Table 12.

At different speeds, 1D-CNN achieves high accuracy rate in road surface recognition. For traditional recognition algorithms, differences in peak-to-peak values and waveform steepness generated by road surface excitation at different speeds can affect the recognition accuracy rate of traditional algorithms. However, 1D-CNN, with its unique automatic feature extraction capabilities, can adaptively learn and capture these changing features. It can perform road surface recognition tasks stably and accurately, further verifying the effectiveness and robustness of 1D-CNN in road surface recognition applications.

### 4.3. Ablation Study

To quantitatively assess the contributions of data augmentation, Adam optimization, and *L*2 regularization, a comprehensive ablation study was conducted. The baseline model was the 1D-CNN architecture proposed in this paper, trained using the SGDM optimizer and without these three components. Each component was then added sequentially, and the corresponding accuracy rate on the same test set was recorded. The results are shown in Table 13.

The experimental results show that the performance of the baseline model (80.67%) confirms the existence of overfitting. The final complete model, which includes all components, achieved an accuracy rate of 99.33%, significantly outperforming traditional methods, thereby demonstrating the effectiveness of our overall design. Experiment four shows that *L*2 regularization is the most effective single component for suppressing overfitting, delivering a substantial 15.00% improvement when used alone. This indicates that our model architecture has sufficient capacity, and that *L*2 is crucial for constraining its complexity and enhancing its generalization capabilities. Experiments three and five reveal the complexity of the Adam optimization algorithm. When used alone, its efficient optimization capabilities slightly exacerbate overfitting (Experiment three). When combined with data augmentation but without *L*2 constraints, its negative effects outweigh the benefits of data augmentation, leading to performance degradation (Experiment five). This demonstrates that Adam requires a robust regularization algorithm to constrain it. Experiment six achieved an accuracy rate of 99.00%, demonstrating that *L*2 regularization effectively “tames” Adam, enabling it to converge rapidly to a solution with exceptional generalization capabilities. Experiment seven achieved an accuracy rate of 98.00%, with the combined effect of the two being superior to either used alone (84.67%/95.67%), proving their complementarity. Experiment eight (full model) achieved the highest performance of 99.33%, indicating that data augmentation provides unique, additional performance gains on top of the powerful combination of Adam and *L*2.

## 5. Conclusions

This paper designs a vibration signal acquisition system based on vehicle vibration signals and achieves accurate identification of different road surface types using an improved 1D-CNN algorithm. The test results show that the developed vibration signal acquisition system can stably obtain road surface vibration data, enabling the acquisition, storage, and transmission of vibration signals when vehicles travel on different road surfaces. The improved 1D-CNN algorithm demonstrated excellent recognition performance in both public datasets and actual vehicle tests, achieving recognition accuracy rates of 99.3% and 99.4%, respectively, significantly outperforming traditional methods. The test also demonstrates that the vibration data from the front brake discs most accurately reflected road conditions due to the short transmission path and minimal interference. In addition, the algorithm maintains high robustness across different vehicle speeds, verifying its applicability under complex operating conditions.

This study provides reliable technical support for vehicle maintenance and road condition monitoring, verifies the potential of road identification methods based on vibration signals in practical applications, and has important engineering application value. In the era of artificial intelligence, autonomous driving technology and robotics are developing rapidly, and road surface recognition technology has become a key pillar in the field of environmental perception. Future work may further explore the application of road surface recognition technology in the field of autonomous vehicles, which can be considered from the following three aspects: First, we will expand the dataset by adding complex road surface types, such as wet and icy roads, and increasing vehicle speeds to enhance the algorithm’s adaptability to different scenarios. Second, the algorithm structure can be further optimized to design a more lightweight network structure. For example, consider combining the MobileNet series architecture with 1D-CNN to achieve millisecond-level response times for road surface recognition technology in unmanned areas. We can also attempt to train a model that uses speed and vibration signals as joint inputs, enabling the model to adapt to varying speeds. Third, explore the deep integration of road surface recognition algorithms and autonomous driving control systems to achieve closed-loop control of “Perception-Decision-Execution.”

## Figures and Tables

**Table 1:** Sensor parameter table.

Parameters	Value
Sensitivity (±10%)	50.1 mV/g
Range	±100 g pk
Resolution ratio	1 mg rms
Frequency response range (±10%)	0.5~10 kHz
Resonant frequency	30 kHz
Excitation voltage	+18~+28 VDC
Excitation current	2~10 mA
Operating temperature range	−40~+121 °C
Weight	20 g

**Table 2:** Main chip selection table.

Main Modules	Chip Model
MCU main control module	GD32F470VIT6
A/D conversion module	CL1606
eMMC storage module	FEMDMW016G
Ethernet communication module	Integrated MAC controller
	YT8512H (PHY chip)
	H1102NLT (transformer)
Clock module	SD2406API
Inertial navigation module	WTGPS-300
Power supply module	SGM6601YTN5G/TR (URB2405S/AMS1117/DB628/B0505S)

**Table 3:** Functions table.

Main Functions	Explanation
Void screenInit();	Interface initialization
Void connectionInit();	Signal and slot connection initialization
Void addTextInformation(QString note);	Add information to the information column
Void fillTabWidget(QStringList fileName, QStringList fileList);	Fill in the file information form
Void slots_DealData(QByteArray receiveData);	Data processing
Void slots_Udpwrite(QByteArray sendarray);	Send data
Void slots_UdpRead();	Receive data

**Table 4:** Detailed architecture of the lightweight 1D-CNN.

Layer Number	Layer Type	Kernel Size/Stride	Number of Kernels	Output Size
1	Input Layer	-	-	20,000 × 1
2	Convolution 1	3 × 1/1	3	20,000 × 3
3	Max Pooling 1	2 × 1/2	-	10,000 × 3
4	Convolution 2	3 × 1/1	3	10,000 × 3
5	Max Pooling 2	2 × 1/2	-	5000 × 3
6	Convolution 3	3 × 1/1	8	5000 × 8
7	Max Pooling 3	2 × 1/2	-	2500 × 8
8	Convolution 4	3 × 1/1	8	2500 × 8
9	Max Pooling 4	2 × 1/2	-	1250 × 8
10	Convolution 5	3 × 1/1	8	1250 × 8
11	Max Pooling 5	2 × 1/2	-	625 × 8
12	Flatten Layer	-	-	5000
13	Fully Connected	-	20	20
14	Output Layer	-	3	3

**Table 5:** Public dataset road surface sample data volume and corresponding labels.

Road Surface Type	Number of Training Set Samples	Number of Test Set Samples	Label
dirt road	400	100	1
cobblestone road	400	100	2
asphalt road	400	100	3

**Table 6:** The performance of different algorithms.

Classification Algorithm	Data Class	Accuracy Rate	F1 Score	Precision	Recall
KMC	Asphalt Road	60.42%	75.40%	64.22%	91.31%
	Cobblestone Road		52.41%	51.11%	53.77%
	Dirt Road		35.92%	60.81%	25.49%
SVM	Asphalt Road	72.68%	95.05%	92.82%	97.38%
	Cobblestone Road		55.64%	47.22%	67.71%
	Dirt Road		54.37%	70.37%	44.30%
KNN	Asphalt Road	74.79%	95.97%	97.33%	94.65%
	Cobblestone Road		53.65%	51.65%	55.80%
	Dirt Road		65.39%	66.12%	64.68%
LSTM	Asphalt Road	92.73%	98.52%	98.83%	98.21%
	Cobblestone Road		85.46%	86.43%	84.51%
	Dirt Road		90.49%	89.44%	91.57%
CNN	Asphalt Road	93.17%	98.96%	99.00%	98.93%
	Cobblestone Road		85.84%	89.23%	82.71%
	Dirt Road		90.85%	88.59%	93.24%
CNN-LSTM	Asphalt Road	92.77%	98.62%	98.46%	98.77%
	Cobblestone Road		85.56%	90.69%	80.98%
	Dirt Road		90.22%	87.20%	93.46%
1D-CNN	Asphalt Road	99.33%	98.99%	98.99%	98.99%
	Cobblestone Road		99.06%	99.06%	99.06%
	Dirt Road		100.00%	100.00%	100.00%

**Table 7:** Frequency table for checking data set category imbalance.

Label	Number of Training Set Samples	Percent	Number of Test Set Samples	Percent
1	405	33.75%	95	31.67%
2	394	32.83%	106	35.33%
3	401	33.42%	99	33.00%

**Table 8:** Fold cross-validation results table.

Fold	Training Set Accuracy Rate	Test Set Accuracy Rate	Difference
1	99.58%	93.67%	5.91%
2	99.92%	95.00%	4.92%
3	100.00%	97.67%	2.33%
4	100.00%	97.67%	2.33%
5	100.00%	98.33%	1.67%
Average	99.90%	96.47%	3.43%

**Table 9:** Noise test results.

Noise Intensity	SNR (dB)	Accuracy Rate	Drop Rate
0.00	∞	99.3%	0.00%
0.05	32.8	99.00%	−0.30%
0.10	26.8	98.33%	−0.97%
0.20	20.8	92.67%	−6.63%
0.50	12.8	72.33%	−26.97%
1.00	6.8	61.33%	−37.97%

**Table 10:** Actual road sample data volume and corresponding labels.

Road Surface Type	Number of Training Set Samples	Number of Test Set Samples	Label
cement road	560	140	1
red brick road	560	140	2
asphalt road	560	140	3
stone-paved road	560	140	4
dirt road	560	140	5

**Table 11:** Accuracy rate of key areas recognition at the same vehicle speed.

Key Areas	Accuracy Rate
battery box	98.9%
rear wheel suspension	99.0%
front wheel brake disc	100.0%

**Table 12:** Accuracy rate of front wheel brake disc recognition at different speeds.

Different Speeds	Accuracy Rate
10 km/h	100.0%
20 km/h	99.9%
30 km/h	99.4%

**Table 13:** Results of the ablation study.

Experiment Name	Accuracy Rate	Enhancement	Training Time
Baseline Model	80.67%	-	60.96 s
Baseline + Data Augmentation	84.67%	+4.00%	56.38 s
Baseline + Adam	79.33%	−1.34%	58.22 s
Baseline + *L*2	95.67%	+15.00%	57.16 s
Baseline + Data Augmentation + Adam	78.33%	−2.34%	59.06 s
Baseline+ Adam + *L*2	99.00%	+18.33%	60.64 s
Baseline+ Data Augmentation + *L*2	98.00%	+17.33%	57.68 s
Full Model	99.33%	+18.66%	60.26 s

## Data Availability

Data is contained within the article.
